# Evaluating the Perceptions, Expectations, and Concerns of Community Pharmacists in Germany Regarding Prescribing by Pharmacists

**DOI:** 10.3390/healthcare13192490

**Published:** 2025-09-30

**Authors:** Niklas Zimmermann, Jan Siefert, Trudi McIntosh, Ágnes Mészáros

**Affiliations:** 1 Department of Pharmacy Administration, Faculty of Pharmacy, Semmelweis University, Hőgyes Endre u. 7-9, 1092-H Budapest, Hungary; 2 School of Pharmacy and Life Sciences, Robert Gordon University, Garthdee Road, Aberdeen AB10 7GJ, UK

**Keywords:** non-medical prescribing, pharmacist prescribing, pharmacy, prescribing models, healthcare access, survey

## Abstract

**Background**: Expanding the role of healthcare professionals could improve patient care. Workforce shortages and financial challenges within the healthcare system have led to non-medical prescribing models, such as pharmacist prescribing in several countries. However, in Germany, the prescribing authority remains with medical professionals, leaving pharmacist prescribing unexplored. **Objectives**: The aim of this study was to assess community pharmacists’ perceptions of the concept of prescribing by pharmacists, to investigate general concerns, and to evaluate the overall readiness and willingness of community pharmacists to take on prescribing responsibilities. **Methods**: A cross-sectional anonymous online survey of community pharmacists was conducted between August and October 2023. The 22-item questionnaire included demographic characteristics, daily work routines, and statements regarding the concept of prescribing by pharmacists. The data were coded and analyzed using IBM SPSS Statistics version 28.0.1. **Results**: The key finding was that attitudes toward prescribing by pharmacists were generally very positive, regardless of the pharmacists’ age and the size of the city in which the pharmacy was located. This positive perception was reflected by 82.3% of the participants, indicating support for the concept of prescribing by pharmacists in Germany. Additionally, 81.8% expressed confidence in taking on a prescribing role after completing the necessary qualifications and training, which 85.9% of all participants considered a basic requirement. **Conclusions**: Most participants expressed strong support for prescribing by pharmacists and indicated openness to its implementation on a certain scale while addressing the importance of further and specialized training.

## 1. Introduction

According to the World Health Organization [1], to ensure the quality of patient care, every quality healthcare service must be “timely”, “equitable”, “integrated”, and “efficient.” The successful introduction and development of non-medical prescribing in various countries is a valid example of how the healthcare system can improve access to medicines and patient care [2,3]. Non-medical prescribing (NMP) refers to the prescription of medicines by healthcare professionals who are not doctors or dentists and who have completed an approved course [4]. The requirements vary from country to country.

Graham-Clarke et al. [5] examined NMP in the United Kingdom (UK) National Health Service in 2019 as part of a systematic policy review; the authors concluded that government policy on NMP has changed since 2006. Through the continuous development of NMP models since 2006, the number of professions whose members are allowed to complete training as non-medical prescribers has increased significantly. The authors concluded that the development of NMP has led to changes in patient care. NMP not only allows patients to be treated by the best-qualified professional but also creates the opportunity for a single healthcare professional to provide patients with a comprehensive package of care. Furthermore, emphasis is placed on alleviating the workload of doctors and reducing costs by minimizing redundant appointments and optimizing the use of resources [2,5].

Prescribing by pharmacists, a subset of NMP, is gaining importance worldwide. In the UK, pharmacists have been authorized to prescribe medications since 2003 through a supplementary prescribing model, in which they collaborate with an independent prescriber (IP) and follow a clinical management plan [6,7]. Since 2006, they have also been legally empowered to prescribe independently, taking full responsibility for patient assessment and prescribing decisions [8].

Pharmacists in the UK prescribe medications in a wide variety of settings for a wide range of conditions, across primary and secondary care, and particularly in community pharmacies, which is relevant to the current study. Two examples of models for the treatment of minor illnesses by community pharmacists (CPs) are the “Pharmacy First” initiative in England and “Pharmacy First Plus” in Scotland. Within the regulatory framework in England, CPs have specific rights that authorize them to prescribe and dispense a limited range of prescription medications for the treatment of seven common illnesses [9,10].

Karim et al. [11] focused on independent prescribing in community pharmacies in Scotland. The authors concluded that participants who independently prescribed were generally very positive about the integration of pharmacist prescribing in community pharmacies.

Unlike in the UK, Poland currently has only one prescribing model by pharmacists. Since 2002, CPs in Poland have been granted limited authorization to prescribe medications, specifically for emergencies [12]. In March 2020, pharmacists in Poland were granted extended authorization to continue existing therapies, allowing them to issue prescriptions for patients, family members, and themselves [12,13]. Miszewska et al. [14] employed a questionnaire to investigate the use of pharmacist-based prescribing powers in Poland. The study’s findings indicated that 75.62% of pharmacists exercised this authorization.

In addition to the models of prescribing by pharmacists already known in other countries, such as IP and prescribing for minor ailments, a further distinct model was specifically designed for and described in the current study. This model is a more restrictive version of the supplementary prescribing model. The difference from the already-known supplementary prescribing model is that the prescribing pharmacist in the modified model works in a community pharmacy and is only allowed to issue repeat prescriptions based on a clinical management plan. Therefore, a repeat prescription is defined as “a prescription for a medicine that you have taken before or that you use regularly” [15]. Because of the historical separation between the pharmacy and medical professions and the resulting barriers that could potentially interfere with a possible introduction, we assume that a more restrictive prescription model would find greater acceptance and the likelihood of a possible introduction would be higher.

Training models for pharmacists to obtain prescribing privileges varied across the world. In the UK, many universities offer courses that provide the knowledge and skills that, along with a period of supervised learning in practice, are required for pharmacists to obtain prescribing privileges [16]. In 2016, Cope et al. [4] concluded that students aspiring to assume a prescribing role should receive training during their undergraduate studies as this would foster a positive attitude toward prescribing. Recently, the concept of pharmacist education and training has developed significantly, with new standards for the initial education and training of pharmacists, published by the General Pharmaceutical Council, being in place since 2021 [17]. Since then, prescribing education and training have been incorporated into a five-year undergraduate and postgraduate program, enabling registered pharmacists to qualify as independent prescribers upon registration [17].

The advantages and possibilities of prescribing by pharmacists include the following points. Prescribing by pharmacists can improve access to medicines and the healthcare system by providing patients with the medicines they need, especially within a reasonable timeframe [3]. The other advantages of enabling pharmacists to prescribe medications include potential savings and the associated more efficient use of resources in the healthcare system [18,19,20]. This allows physicians to concentrate on difficult, time-consuming emergencies [21]. In the case of minor illnesses, it offers the greatest potential and can ease the financial burden on the healthcare system [22].

The situation in other countries, which have introduced prescribing by pharmacists, is similar to the current situation in Germany. In rural areas, there is a frequent shortage of doctors, and the supply of prescription medicines to the population is only partially secured [23]. Recently, regulations governing the prescribing of medications in various countries have mirrored the stringent standards observed in present-day Germany, where only licensed medical professionals, such as doctors and dentists, possess the authority to issue prescriptions [24,25]. In the healthcare industry, prescribing medication is a multistage process [26], with an authorized person issuing the patient with a written or digital prescription for a specific medication. With the prescription, the patient is authorized to obtain the medication at a licensed pharmacy for personal use. In Germany, medicines are categorized into different groups: prescription-only medicines and nonprescription medicines, which are commonly known as over-the-counter (OTC) medications. Nonprescription medicines are further divided into pharmacy-only and general-sale medicines [27].

One example of how strict prescribing regulations are becoming less stringent in Germany is the proposed Nursing Competence Act, which is currently being discussed. It is intended to give nurses extended powers, similar to Advanced Nurse Practitioners in the UK or Advanced Practice Registered Nurses in the US. These advanced practice nurses are authorized to prescribe medications within their area of competence after completing extended and intensive training, including postgraduate nursing and prescribing training [28,29,30]. In 2023, German healthcare politicians emphasized their support for the Nursing Competence Act, indicating political readiness for NMP, which might be the first step toward implementing NMP [31].

The COVID-19 pandemic has highlighted the essential role of community pharmacies in providing crucial patient care. Because of the nationwide distribution and easy access to pharmacies, pharmacies were able to maintain the supply of medicines even during social distancing and lockdowns [32]. Based on the success of COVID-19 vaccination in pharmacies, German legislators have recognized the provision of vaccinations as a standard pharmacy service in Section 1a (11) of the Pharmacy Operating Regulations (Apothekenbetriebsordnung) [33]. This illustrates the appreciation of pharmacists’ health literacy and their increased involvement in primary care. A new advancement in broadening the scope of pharmacists’ skills involves the implementation of “pharmaceutical services” within Germany. The Pharmacy Strengthening Act (Vor-Ort-Apotheken-Stärkungsgesetz) in Germany has given pharmacies the opportunity to offer remunerated services to pharmacy customers to improve patient compliance for the first time [34]. Two important competence-enhancing pharmaceutical services that should be mentioned here are medication advice for patients with polypharmacy and education on adequate inhaler techniques [34]. Thus, the scope of CPs’ activity has evolved and no longer relates exclusively to the traditional task of “ensuring the proper supply of medicines to the population in the public interest”, as set out in Section 1 (1) of the German Pharmacies Act (Apothekengesetz) [35]. Another example that demonstrates the topical relevance of this subject and illustrates that the expansion of pharmacists’ roles in Germany has only just begun is the position paper published by the German Federal Union of German Associations of Pharmacists (Bundesvereinigung Deutscher Apothekerverbände) on 10 April 2025, entitled “Towards a Healthy Future with the Pharmacy” (In eine gesunde Zukunft mit der Apotheke). In this document, the authors emphasize the high level of professional competence within the pharmacy profession and therefore propose a further expansion of pharmacists’ roles. The suggested extensions of competence include, for example, broadening the scope of care provided during night and emergency services, as well as expanding vaccination and screening services. The primary objectives are clearly defined as “more direct access to medicines”, “prevention and early detection”, and “greater support for safe pharmacotherapy” [36].

In Germany, the adaptation of the pharmacy degree program is currently under discussion. In line with this, the general meeting of the Federal Chamber of Pharmacists (Bundesapothekerkammer) adopted a position paper on 10 May 2022 [37]. A central demand is to extend the pharmacy degree program by two semesters to address the evolving roles of pharmacists. Additionally, future clinical training should focus more on “patient and drug therapy safety” and “evidence-based therapy support.” In this context, the teaching of clinical pharmacy is to be particularly intensified.

The current healthcare policy [38] and structural deficits in nationwide patient care highlight the urgent need for a systemic change in the German healthcare system [39]. The expansion of pharmacists’ competencies is only beginning to drive this change.

A review of the literature at the time of study design and implementation indicated that no studies had investigated the concept of pharmacist prescribing and its potential implementation in Germany, highlighting a significant research gap in this area. There is a need to explore prescribing by pharmacists as a viable and globally recognized approach to improving the accessibility of patient care to address the current lack of understanding in this area. This study is part of a broader research initiative aimed at capturing perspectives on prescribing by pharmacists from multiple stakeholder groups in Germany. While the present study focuses on CPs, a related study surveying pharmacy customers was conducted in parallel and has recently been published [40]. Together, these studies provide complementary insights into the broader discourse surrounding the potential implementation of prescribing by pharmacists. The effectiveness of any new practice or transformation heavily relies on the acceptance and willingness of the target professional group to implement it. Given international developments and the gap in Germany, this study aims to evaluate CPs’ perceptions, expectations, and concerns regarding a potential pharmacist prescribing role in Germany.

## 2. Materials and Methods

An online survey was used to investigate CPs’ perceptions, expectations, and concerns regarding prescribing by pharmacists in Germany for the first time. This study, which was conducted between August and October 2023, involved pharmacists working in community pharmacies in Germany.

### 2.1. Questionnaire Development

In the current study, the questionnaire was designed to be appropriate for the German healthcare system and to ensure comprehensive coverage of topics related to the concept of prescribing by pharmacists. The questionnaire comprised 22 questions, which were separated into two distinct sections: The first section gathered information about the demographic characteristics of the participating pharmacists, such as their age, gender, professional status, and years of experience. Additionally, the study delved into aspects of the participants’ daily work routine within the community pharmacy setting. The criteria for recommending OTC medications were developed with reference to the “Ten Principles of Good Prescribing” [41] and adapted to the OTC context. The second section assessed the participants’ perceptions, expectations, and concerns regarding the proposed expansion of pharmacists’ roles and responsibilities. This was achieved by measuring their level of agreement using a 5-point Likert scale with a total of 13 attitude statements, covering topics such as confidence in assuming a prescribing role, training needs, patient safety, potential benefits, and other professional aspects In addition to the Likert scale items, the questionnaire included both single- and multiple-choice questions.

The questionnaire was piloted in a paper form with 24 pharmacists to test for face and content validity. Face and content validity were also ensured through separate evaluations by the study’s authors, followed by expert review conducted by two specialists from Semmelweis University. Following the pilot study, minor adjustments were made to the questionnaire to ensure clarity and comprehensibility, particularly in terms of the rewording of certain questions. No questions were removed or added, and the overall structure and content of the questionnaire remained unchanged. A “no answer” response option was intentionally included in the questionnaire to enhance the accuracy and reliability of the collected data. To submit the completed questionnaire, participants were required to provide a response to every question, either by selecting one of the substantive answer options or the “no answer” option. Consequently, only fully completed questionnaires were included in the analysis, and no items had missing data that required further handling. When designing the final version of the questionnaire, care was taken to ensure that the responses could be recorded anonymously to prevent the collection of personal data. The questions on the demographic characteristics of the participants were deliberately designed to ensure that it was not possible to link the answers to a specific individual. The extensive settings options of the LimeSurvey platform regarding data protection and anonymity allowed the survey to be configured so that neither the IP address of the participants nor any other personal data were recorded [42]. The LimeSurvey platform meets the highest data protection standards because it is compliant with the General Data Protection Regulation for European users [42].

### 2.2. Data Collection

To obtain a nationwide picture of the opinions of CPs, different approaches were used to collect data. All email addresses were collected individually from the publicly accessible websites of the pharmacies. Through this method, the email addresses of 390 pharmacies within the Linda Group, the largest pharmacy group in Germany, as well as 3000 other pharmacies in the federal states of Bavaria, Baden-Württemberg, Hesse, Schleswig-Holstein, Saarland, Lower Saxony, Mecklenburg-Vorpommern, North Rhine-Westphalia, and Saxony-Anhalt, were identified. All pharmacies were invited by email to participate in the online survey on the LimeSurvey platform via a provided link and QR code. To maximize reach among CPs, the survey was also disseminated through social media platforms, such as LinkedIn and Facebook. Because the survey was conducted across Germany and targeted a broad group of pharmacists without using a predefined sample list (e.g., invitations shared via social media), the response rate could not be calculated. According to the report “The Pharmacy: Figures, Data, and Facts 2024” (Die Apotheke: Zahlen, Daten und Fakten 2024) by the Federal Union of German Associations of Pharmacists (Bundesvereinigung Deutscher Apothekerverbände) [43], there were 53,178 CPs in Germany in 2023. Using an online sample size calculator [44] with a 95% confidence level, a population size of 53,178, and a 5% margin of error, the ideal sample size was calculated to be 384.

### 2.3. Ethical Issues

The present study was a cross-sectional survey based on a fully anonymized questionnaire, in which no personal data were collected, and no interventions were conducted on participants. The full confidentiality and anonymity of the participants were maintained during the study. Prior to the survey, participants were informed about the study’s purpose, data use, the right to withdraw, their general rights as participants, and that no risks were involved in participating. Participants provided their voluntary and informed consent electronically on the first page of the LimeSurvey questionnaire by selecting “Agree.” After providing their consent, the participants were directed to the main questionnaire. In June 2023, ethics committee approval was waived due to the circumstances outlined in the ethics evaluation document.

### 2.4. Statistical Analysis

The responses from the pilot study were excluded from the final data analysis because the wording of the questions was modified during the pilot study, which could have affected the responses. Data obtained from the questionnaires were exported to the Software Package IBM SPSS Statistics (IBM SPSS 28.0.1.) and analyzed using descriptive statistics. To enhance interpretation and simplify the analysis, the response options were categorized as necessary. For example, to assess the overall positive attitude, the Likert-type scale response options “somewhat agree” and “fully agree” were combined into a single category. All subgroup analyses in this study were conducted using the Mann–Whitney U test, a nonparametric method for ordinal data, to compare Likert-scale response distributions between two-group demographic subgroups, including age, gender, and professional status, and the city size of the pharmacy’s location. The threshold for statistical significance was set at a *p*-value of 0.05.

## 3. Results

At the end of the survey period, 356 participants completed the questionnaire in full. The demographic characteristics of the participants are presented in Table 1. The majority of participants were under 50 years of age (62.3%; n = 222), and 55.3% (n = 197) were female. Compared with pharmacists who currently worked as employees (34.0%, n = 121), nearly twice as many were pharmacy owners (52.8%; n = 188), and 47 (13.2%) were branch managers of community pharmacies. Of the participants surveyed, 11.2% (n = 40) worked in pharmacies located in villages with fewer than 5000 inhabitants, 25.6% (n = 91) in medium-sized cities with populations between 20,000 and 100,000 inhabitants, and 32.0% (n = 114) in larger cities with populations exceeding 100,000. Additionally, 41.9% (n = 149) of the participating pharmacists had over 20 years of professional experience, whereas 24.4% (n = 87) had less than 5 years of experience.

Recommending OTC medications to patients as part of the pharmaceutical consultation service for minor illnesses is a fundamental aspect of pharmacists’ daily practice. The most important factors influencing this activity, according to the participants, are presented in Table 2. The patient’s current complaints, medical history, and potential limits of self-medication (type, severity, and duration of symptoms as well as age) were selected most frequently (95.8%; n = 341). The patient’s medication regimen, including allergies and intolerances, was the second most frequently selected factor, followed by patient’s individual needs and preferences when selecting the most suitable dosage form.

**Figure 1:**
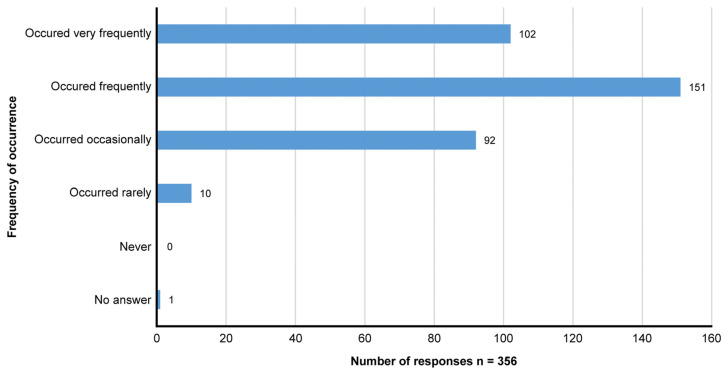
Frequency with which patients presented at the pharmacy, having run out of medication for their long-term therapy. The y-axis represents the frequency of occurrence, and the x-axis shows the number of community pharmacists reporting each occurrence. Total responses: n = 356.

Pharmacists often face the challenge of patients running out of medication for their long-term therapy due to delays in receiving prescriptions, leading to treatment interruptions. A closer inspection of Figure 1 indicates that a large majority of pharmacists (71.1%; n = 253) reported that this situation occurs either frequently or very frequently. Ten (2.8%) pharmacists stated that the situation had rarely occurred, whereas no pharmacists stated that this situation had never occurred.

The second part of the survey focused on assessing the participants’ perceptions, expectations, and concerns regarding the concept of prescribing by pharmacists. First, the pharmacists were asked to select from three different models of prescribing by the pharmacist and state which they would prefer for possible future introduction. Multiple responses were allowed, and the results are summarized in Table 3. A large majority of participants (83.1%; n = 296) preferred the model in which pharmacists were allowed to prescribe medication (repeat prescriptions) based on a predetermined clinical management plan for a medically diagnosed condition. The second most preferred model was the one in which pharmacists were allowed to prescribe certain prescription drugs for certain illnesses or patient groups without a prior medical consultation. Participants’ least-preferred model involved independent prescribing without restrictions or prior medical consultation.

Several statements regarding prescriptions by pharmacists were used to obtain a more in-depth understanding of the pharmacists’ attitudes. The levels of agreement with these statements were measured using a 5-point Likert scale (Table 4). A large majority of pharmacists (81.8%; n = 291) trust themselves to take on a prescribing role to a certain extent after acquiring relevant additional qualifications. There was also broad agreement with the statements regarding the further training of pharmacists. A total of 75.2% (n = 268) of the participants believed that they should enhance their clinical assessment skills before assuming prescribing responsibilities. The Mann–Whitney U test revealed that significantly more participants under 30 years old favored enhancing their clinical assessment skills compared with their older colleagues (50–59 years old) (z = −2.283, *p* = 0.022). The importance of further training and the acquisition of corresponding additional qualifications was recognized by 85.9% (n = 306) of participants as they consider it a basic prerequisite for CPs to undergo further training in line with their prescribing authorization. A majority (83.7%; n = 298) of participants responded with “somewhat agree” or “strongly agree” to the statement, “If pharmacists were allowed to issue prescriptions for medicines for long-term therapy, patients would prefer to have them issued by their CPs.”

The statement that health insurance companies should cover the costs of medications prescribed by pharmacists received the highest level of agreement, with 96.1% (n = 342) of participants “somewhat agreeing” or “fully agreeing.” An important aspect to consider when pharmacists are authorized to prescribe medications is the safety of patients.

The majority of pharmacists also recognized the opportunities for improving patient care that prescribing by pharmacists could offer. A high proportion (91.6%; n = 326) of pharmacists agreed that if prescribing pharmacists were introduced, access to prescriptions for minor illnesses or repeat prescriptions could be made easier with the help of the nationwide pharmacy network and the proximity of pharmacies. Furthermore, 87.4% (n = 311) of the participants fully or somewhat agreed that “the introduction of prescribing pharmacists could relieve the burden on general practitioner (GP) practices. (More time and resources for in-depth medical care of patients would be available in GP practices.)”

The generally positive attitude of the participants toward the topic of prescribing by pharmacists was also reflected in their level of agreement with the final statement, with 82.3% (n = 293) of the participants either “fully agreeing” or “somewhat agreeing” that they liked the concept of prescribing by pharmacists and could envisage its introduction in Germany to some extent. Attitudes did not significantly differ by participants’ age or pharmacy location size (all *p* > 0.05). To analyze whether pharmacy owners and employed pharmacists had different attitudes toward prescribing by pharmacists, branch managers and employed pharmacists were combined into a single group, which was subsequently compared with pharmacy owners. The analysis concluded that although both groups held a positive attitude toward the concept of prescribing by pharmacists, there were no statistically significant differences between the two groups (*p* > 0.05). Gender variations were observed in the level of agreement between the two statements. Male participants expressed greater confidence in taking on a prescribing role after acquiring additional qualifications (z = −3.129, *p* = 0.001). The responses to the statements “I think the concept of prescribing by pharmacists is a good one” and “I can imagine it being introduced in Germany” also revealed statistically significant differences between men and women. While both genders were generally positive in answering these questions, men were statistically significantly more positive (z = −3.219, *p* = 0.001).

The next section addresses possible concerns regarding CPs. In response to the statement regarding potential misuse of prescriptions by pharmacists, 28.1% (n = 100) of the participants considered this a possibility, 24.7% (n = 88) gave a neutral response, and 45.5% (n = 162) expressed no concern regarding potential misuse.

When asked about general concerns regarding the concept of prescribing by pharmacists, 65.7% (n = 234) of participants cited a lack of clarity about the legal framework as the most frequent concern. Concerns about increased bureaucracy because of delays by health insurance companies were the second most common concerns, as mentioned by 49.7% (n = 177) of the participants. Among the predefined concerns, 39.3% (n = 140) of the participants selected the possibility of harm to the patient as a result of a misjudgment, whereas 16.6% (n = 59) expressed concerns that the quality of care would not correspond to that of a doctor if pharmacists were allowed to prescribe independently. The response option “Other” was selected by 19.1% (n = 68) of the participants. No concerns were expressed by 16% (n = 57) of the participants (Table 5).

## 4. Discussion

To the best of our knowledge, this is the first survey to explore how CPs in Germany view the potential implementation of pharmacist prescribing. It provides novel insights and offers an initial assessment of their perceptions, expectations, and concerns. Additionally, this study sought to establish a foundation for further research in this area.

The key finding of our study was that most pharmacists expressed a highly positive perception across all statements regarding the concept of prescribing by pharmacists, highlighting their willingness to take on new professional roles. Data highlighted positive attitudes toward prescribing pharmacists among participating pharmacists from all sizes of pharmacy locations, indicating broad acceptance and openness to evolving roles within the pharmacy profession. The professional status of CPs, comprising “owner of a community pharmacy”, “branch manager of a community pharmacy”, and “employed pharmacist in a community pharmacy”, had minimal influence on the preference for the introduction of prescribing pharmacists. Another key finding of the current study was that most participants believed that additional training should be a fundamental prerequisite for pharmacists before they are authorized to prescribe. Furthermore, the participants indicated a need to enhance their clinical assessment skills before prescribing rights being granted. In the context of the potential future implementation of prescribing by pharmacists, several important concerns were highlighted, particularly regarding legal frameworks and professional boundaries, as well as a potential increase in bureaucratic workload.

The potential benefits associated with the introduction of prescribing pharmacists in Germany received high approval ratings from the pharmacists surveyed. CPs therefore anticipated that the introduction of pharmacist prescribing offers the potential to improve access to repeat prescriptions and prescriptions for minor ailments, as well as to alleviate the burden on GP practices.

Rafferty et al. [19] analyzed a Canadian model of pharmacist prescribing medications for minor ailments. The authors concluded that this model could result in cost savings and enhance access to healthcare. The authors also suggested that this model could be a promising option for implementation in other countries where no similar pharmacist prescribing model for minor ailments currently exists.

Switzerland introduced a pharmacist prescribing model for managing minor ailments in 2019, aiming to limit medical consultations and reduce healthcare costs. Amador-Fernández et al. [45] analyzed the clinical relevance of the Swiss model and found that the current list of medicines approved for pharmacist prescription is inadequate. This is because most prescription drugs on the list have an OTC equivalent that patients can obtain without using a prescription service. The authors highlighted an important aspect in the potential introduction of a similar model elsewhere, namely, that the selection and composition of medicines approved for pharmacists to prescribe play a crucial role in achieving the intended objectives.

In the current study, a large majority of the participants agreed with the statement that “if pharmacists are allowed to issue prescriptions for drugs for long-term therapy, patients would be more likely to have them issued by their CPs.” Rodriguez et al. [46] investigated women’s reasons and experiences of obtaining contraceptives from a prescribing pharmacist in the USA, and their findings were similar to ours; the “convenient location” of the pharmacy and ease of access were cited as reasons why women tended to obtain their contraceptives from a pharmacist.

Additionally, this study addressed the question of which prescribing-by-pharmacist model would be preferred by German CPs for potential implementation. The results showed a clear preference for the model in which pharmacists would issue repeat prescriptions for chronic conditions based on a clinical treatment plan. The frequency at which patients ran out of medication for long-term therapy due to not receiving prescriptions in time was also identified as a common and significant issue. This may explain the participants’ support for the prescribing pharmacist model, which would allow them to issue repeat prescriptions based on a clinical management plan. Jebara et al. [21] examined stakeholders’ views and experiences regarding pharmacist prescribing in a systematic review. The authors identified challenges related to diagnosis and liability issues regarding independent prescribing as the main reasons why some pharmacists preferred to prescribe medications for minor illnesses or long-term conditions.

In addition to the need to enhance the quality of clinical assessment skills, participants identified the necessity for further training and education, tailored to the relevant prescribing model, as a fundamental prerequisite for obtaining prescribing authorizations. The need for extensive additional training was recognized in other studies. Cooper et al. [47] and George et al. [48] conducted research in the early days of pharmacist supplementary prescribing in England and Scotland, respectively, reflecting the initial stages following the introduction of pharmacists’ prescribing authorization. The circumstances at that time are comparable to those of the proposed system for the German healthcare framework.

Cooper et al. [47] examined the perceptions of course participants and their subsequent application in practice. A majority of 62% indicated that they had acquired the requisite prescribing skills, whereas 82% found the courses to be useful. Furthermore, George et al. [48] surveyed CPs on the subject of independent prescribing and found that 97.7% of pharmacists expressed confidence in their ability to independently prescribe, whereas 88.4% of participants considered clinical training related to medications to be important.

Training and practice have undergone significant development since these studies were conducted in the UK. In a systematic review of the expertise development of independent pharmacists and nurse prescribers in the UK, Abuzour et al. [49] concluded that continuous practice in the workplace can enhance skills.

Woit et al. [50] showed in their scoping review that although pharmacists are capable of prescribing drugs, they lack self-confidence. These findings highlight the need for additional educational initiatives.

The Prescribing Safety Assessment (PSA) is a prescribing skills evaluation tool developed in the UK for medical students with the primary aim of demonstrating proficiency in prescribing and a focus on effective and safe medication use [51]. In a study conducted in Scotland, pharmacist prescribers were assessed and compared with medical students. The performance of pharmacists in the PSA was found to be equivalent to that of medical students [52]. Another study, in which student and preregistration trainee pharmacists undertook the PSA, found that diagnostic skills in particular need to be further improved, as was found in the current study. This suggests that the curriculum for pharmacists in the clinical area should be expanded [51], as has happened in the UK, to allow pharmacists to prescribe independently from the point of registration. If CPs in Germany are to be authorized to prescribe in the future, participants agreed that the current clinical training provided as part of pharmacy studies is not yet sufficient. Therefore, the majority of participants considered specific further and advanced training, adapted to the respective prescribing model, fundamental. Interestingly, the results also indicate that CPs already apply comprehensive patient assessment in their daily OTC counseling (Table 2), which parallels the principles of good prescribing. This existing skillset could be leveraged and further developed through formal prescribing training, providing a valuable foundation for future prescribing responsibilities.

The findings of the current study provide comprehensive insights into participants’ concerns regarding the potential introduction of prescriptions by pharmacists. The most frequently cited concern was the “lack of clarity about the legal framework.” Despite receiving various models in the questionnaire, most participants questioned the feasibility and extent of establishing a legal framework for prescribing by pharmacists in Germany. This uncertainty likely arises from the historical strict separation between medical and pharmacy professions in Germany [53,54] and the fact that German law currently does not permit prescribing by pharmacists. Consequently, significant legislative changes would be required to allow for such practices, which may explain why pharmacists are understandably uncertain about how a prescribing system might be structured. Furthermore, participants expressed concerns about the potential deterioration of the doctor–pharmacist relationship with the introduction of pharmacist prescribers, fearing significant resistance from the medical profession. This resistance was previously observed during discussions about the participation of pharmacies in nationwide COVID-19 vaccination campaigns. In a survey conducted by the German Physician Portal (DeutschesArztPortal) involving 849 participants, 77% of physicians considered that pharmacists should not administer COVID-19 vaccinations [55]. The study findings underscore the importance of engaging the medical profession in the development of pharmacist prescribing models in order to address concerns and prevent inter-professional tension. The third most common concern is the possibility of patient harm because of potential misjudgments by pharmacists in prescribing roles. McIntosh et al. [56], in an early study, explored the views and reflections of undergraduate pharmacy graduates on pharmacist prescribing practices. The findings revealed concerns similar to those identified in our study. For instance, participants expressed apprehension about the impact of inadequate diagnostic skills on their prescribing competence. Additionally, concerns about the emerging professional rivalry between physicians and prescribing pharmacists were noted, which is consistent with the observations in this study.

Through their scoping review, Zhou et al. [57] investigated the barriers to pharmacist prescribing by comparing experiences in the UK, New Zealand, Canada, and Australia. The authors reported that concerns regarding patient safety were highlighted in Canadian studies. These concerns stemmed from the pharmacists’ lack of access to medical records and insufficient diagnostic skills. An important barrier identified was the perceived possibility of conflict of interest when pharmacists assume prescribing and dispensing roles. By comparison, only 28.1% of the participants surveyed in the current study stated that prescriptions could be abused by pharmacists. In contrast, 24.7% of participants were neutral regarding the risk of prescriptions abuse by pharmacists, whereas 45.5% did not perceive abuse as a problem.

The findings of this study demonstrate that the potential introduction of prescribing by pharmacists is associated with several concerns. For successful implementation in the Germany, it will be essential to examine not only the perspectives of pharmacists but also those of other healthcare stakeholders. The results of this study may serve as an initial assessment, highlighting key areas of concern to be addressed, including the establishment of clear regulatory frameworks and the development of appropriate education and training opportunities.

According to the most recent survey conducted by the Federal Chamber of Pharmacists (Bundesapothekerkammer) in 2019, the average age of pharmacists working in community pharmacies was 47.8 years, whereas that of nonpharmacy owners was 45.6 years [43]. The average age of the pharmacists was 43.6 years. Participation in our study was based on self-selection, and the distribution of age groups was acceptable, with the average age of participants being slightly lower than that of the broader statistical survey.

### 4.1. Strengths and Limitations

One notable strength of this research is its innovative approach, as only a few countries have introduced prescribing by pharmacists. Currently, there are no pharmacists in Germany with this authority. Consequently, this study could inform the introduction of prescribing pharmacists in Germany. With data from 356 participants, the sample size is close to the calculated ideal of 384 (margin of error: 5%; confidence level: 95%). This provides valuable insights into the topic of prescribing by pharmacists, with a considerable degree of robustness and reliability.

A potential limitation of this study is the sampling strategy, which may have introduced selection bias and limits the representativeness of the findings. This is particularly relevant given the lack of a calculable response rate. Because invitations were sent to pharmacy contact emails and participation was based on self-selection via social media, pharmacy owners and those particularly interested in the topic may be overrepresented in the sample. In addition, CPs with especially strong opinions may have been more likely to participate, which could have biased the overall positivity of responses. This potential bias is also consistent with a comparison to survey data from the current study with the survey data from the Federal Union of German Associations of Pharmacists (Bundesvereinigung Deutscher Apothekerverbände), which indicated that our study sample included a higher proportion of pharmacy owners and men, as well as a slightly lower average age of pharmacists working in community pharmacies [43]. This is notable because men demonstrated statistically significantly greater support in our study for prescribing pharmacists compared to women. The data were collected as part of a survey conducted in Germany; therefore, they may not be generalizable to other countries.

A further limitation of this study is the categorization of CPs’ professional experience. In particular, the option of 10 years of professional experience could be selected in the 5–10 and 10–20 years categories. This overlap may have resulted in a slight distortion of the data on professional tenure.

### 4.2. Recommendations for Further Research

This study contributes to the literature by exploring novel perceptions, expectations, and concerns of CPs in Germany regarding prescribing by pharmacists. To gain a more comprehensive understanding of the current situation, and to support the future implementation of pharmacist prescribing, further research is needed. This research should also consider the perspectives and concerns of other healthcare professionals, including physicians, as well as national healthcare authorities. Future studies should also investigate which curricular content and training components are necessary to adequately prepare pharmacists for prescribing responsibilities. Additionally, the role of clinical laboratory analyses in supporting supplementary prescribing for chronic patients should be considered. As part of a broader research initiative, a recently published study has already examined pharmacy customers’ attitudes toward pharmacist prescribing [40]. Future research should integrate these complementary perspectives to identify stakeholder alignment, address potential areas of conflict, and inform the development of an appropriate legal framework in collaboration with key decision-makers.

## 5. Conclusions

This study was crucial in addressing the knowledge gap by providing insights into CPs’ perceptions, expectations, and concerns regarding the concept of prescribing by pharmacists and its potential introduction in Germany. The majority of the participants expressed strong support for prescribing by pharmacists, while highlighting the importance of further and specialized training, as well as concerns regarding the legal framework and professional boundaries. The participants indicated support for implementing prescribing by pharmacists in Germany in a limited, well-defined scope, for instance, by permitting repeat prescriptions for chronic medications under a collaborative practice agreement. The participants believed that adopting these new roles could have the potential to improve patient care and reduce the workload of other healthcare professionals. These insights not only pave the way for further research in this area but can also be used by healthcare decision-makers to design feasible pharmacist prescribing models and the necessary training programs, thereby harnessing pharmacists’ willingness to expand their role for the benefit of the healthcare system.

## Figures and Tables

**Table 1:** Demographic characteristics of community pharmacists (N = 356).

Characteristics	n (%)
**Age (years)**	
Less than 30	67 (18.8)
30–39	92 (25.8)
40–49	63 (17.7)
50–59	90 (25.3)
60–69	39 (11.0)
70 and over	4 (1.1)
No answer	1 (0.3)
**Gender**	
Male	158 (44.4)
Female	197 (55.3)
No answer	1 (0.3)
**Professional Status**	
Owner of a community pharmacy	188 (52.8)
Branch manager of a community pharmacy	47 (13.2)
Employed pharmacist in a community pharmacy	121 (34.0)
**City size of the pharmacy’s location**	
Village with less than 5000 inhabitants	40 (11.2)
Small town between 5000 and 20,000 inhabitants	108 (30.3)
Medium-sized city between 20,000 and 100,000 inhabitants	91 (25.6)
Large city with over 100,000 inhabitants	114 (32.0)
No answer	3 (0.8)
**Professional experience as a community pharmacist (years)**	
Less than 5	87 (24.4)
5–10	54 (15.2)
10–20	63 (17.7)
More than 20	149 (41.9)
No answer	3 (0.8)

**Table 2:** Factors that impact OTC selection decision-making.

Recommendation Factors	n (%) *
Taking history of current and past medical complaints, considering the limits of self-medication (type, severity and duration of symptoms and the patient’s age)	341 (95.8)
Consideration of the patient’s complete medication regimen (including allergies and intolerances)	307 (86.2)
Consideration of the patient’s individual needs and preferences when selecting the most suitable dosage form	293 (82.3)
Consideration of patients’ health literacy (including concerns and expectations)	196 (55.1)
Cost–benefit analysis of the respective preparations	123 (34.6)
Benefit-risk assessment of the respective preparations	196 (55.1)
Consideration of the guidelines	171 (48.0)

* Percentages are based on the total number of participants (N = 356), allowing for multiple responses.

**Table 3:** Preferred models of prescribing by pharmacists.

Preferred Prescribing Models	n (%) *
Pharmacists are allowed to prescribe medications based on a predetermined clinical management plan for a medically diagnosed condition. (Mainly refers to the issuing of prescriptions for drugs for long-term therapy (repeat prescriptions).)	296 (83.1)
Pharmacists are allowed to prescribe certain prescription drugs for specific illnesses or patient groups independently and without a prior medical consultation.	103 (28.9)
Pharmacists are allowed to prescribe medicines without restriction, independently and without prior medical consultation.	18 (5.1)
No answer.	18 (5.1)

* Percentages are based on the total number of participants (N = 356), allowing for multiple responses.

**Table 4:** Statements regarding the topic of prescribing by pharmacists.

	n (%)					
Statements	No Answer	Do Not Agree at All	Somewhat Disagree	Neutral	Somewhat Agree	Fully Agree
I would trust myself to take on a prescribing role to a certain extent after acquiring the relevant additional qualifications.	2 (0.6)	10 (2.8)	23 (6.5)	30 (8.4)	100 (28.1)	191 (53.7)
As a community pharmacist, I would trust myself to make a diagnosis for minor illnesses and prescribe medication based on this.	3 (0.8)	13 (3.7)	49 (13.8)	41 (11.5)	113 (31.7)	137 (38.5)
If pharmacists are allowed to issue prescriptions for drugs for long-term therapy, patients would be more likely to have them issued by their community pharmacists (repeat prescription).	2 (0.6)	4 (1.1)	15 (4.2)	37 (10.4)	116 (32.6)	182 (51.1)
I am confident that as a prescribing community pharmacist, I would prescribe as safely as my clients’ associated GP.	5 (1.4)	13 (3.7)	39 (11.0)	63 (17.7)	110 (30.9)	126 (35.4)
My clinical assessment skills would need to be enhanced before I would be allowed to prescribe medication.	3 (0.8)	9 (2.5)	28 (7.9)	48 (13.5)	129 (36.2)	139 (39.0)
I consider it a basic prerequisite for pharmacists to undergo further training in line with their prescribing authorization.	1 (0.3)	8 (2.2)	14 (3.9)	27 (7.6)	77 (21.6)	229 (64.3)
With the help of the nationwide pharmacy network and the convenience of the pharmacy location, access to prescriptions for minor illnesses or repeat prescriptions could be facilitated by prescribing community pharmacists.	3 (0.8)	5 (1.4)	5 (1.4)	17 (4.8)	100 (28.1)	226 (63.5)
The introduction of prescribing pharmacists could relieve the burden on GP practices. (More time and resources for in-depth medical care of patients would be available in GP practices).	3 (0.8)	5 (1.4)	7 (2.0)	30 (8.4)	90 (25.3)	221 (62.1)
Health insurance companies should cover the costs of medicines prescribed by pharmacists. (As is already the case with the existing supply of medicines).	3 (0.8)	4 (1.1)	1 (0.3)	6 (1.7)	28 (7.9)	314 (88.2)
The introduction of prescribing pharmacists would be a good way for the healthcare system to save money.	8 (2.2)	15 (4.2)	24 (6.7)	81 (22.8)	84 (23.6)	144 (40.4)
The introduction of prescribing pharmacists could have a negative impact on patient safety.	3 (0.8)	98 (27.5)	151 (42.4)	60 (16.9)	37 (10.4)	7 (2.0)
I am of the opinion that there would be no abuse of prescriptions by the pharmacists.	6 (1.7)	20 (5.6)	80 (22.5)	88 (24.7)	89 (25.0)	73 (20.5)
I think the concept of prescribing pharmacists is a good one and I can imagine it being introduced in Germany to a certain extent.	2 (0.6)	15 (4.2)	18 (5.1)	28 (7.9)	113 (31.7)	180 (50.6)

**Table 5:** Concerns regarding the concept of prescribing by pharmacists.

Concerns	n (%) *
Lack of clarity about the legal framework.	234 (65.7)
Additional bureaucratic effort due to delays caused by health insurance companies.	177 (49.7)
The possibility of harm to the patient as a result of a misjudgment.	140 (39.3)
Other	68 (19.1)
The quality of care if pharmacists were allowed to prescribe independently would not correspond to that of a doctor.	59 (16.6)
I have no concerns.	57 (16.0)
No answer	3 (0.8)

* Percentages are based on the total number of participants (N = 356), allowing for multiple responses.

## Data Availability

The original contributions presented in this study are included in the article. Further inquiries can be directed to the corresponding author.

## Abbreviations

The following abbreviations are used in this manuscript:

NMP	Non-medical prescribing
UK	United Kingdom
IP	Independent prescriber
CPs	Community pharmacists
OTC	Over-the-counter
GP	General practitioner
PSA	Prescribing safety assessment
